# *CACNB3* defects are associated with infantile idiopathic nystagmus

**DOI:** 10.1093/braincomms/fcag034

**Published:** 2026-02-07

**Authors:** Christoph Jüschke, Kira Linsel, Marta Owczarek-Lipska, Nicola Brandt, Sarah Zunken, Janine Altmüller, Markus N Preising, Dennis Kastrati, Holger Thiele, Mervyn G Thomas, Peter Nürnberg, Birgit Lorenz, Ulrich Kellner, Anja U Bräuer, G Christoph Korenke, Irene Gottlob, John Neidhardt

**Affiliations:** Human Genetics, School VI—School of Medicine and Health Sciences, Carl von Ossietzky Universität Oldenburg, Oldenburg, Germany; Human Genetics, School VI—School of Medicine and Health Sciences, Carl von Ossietzky Universität Oldenburg, Oldenburg, Germany; Human Genetics, School VI—School of Medicine and Health Sciences, Carl von Ossietzky Universität Oldenburg, Oldenburg, Germany; Division of Human Medicine, School VI—School of Medicine and Health Sciences, Research Group Anatomy, Carl von Ossietzky Universität Oldenburg, Oldenburg, Germany; Research Center for Neurosensory Science, Carl von Ossietzky University Oldenburg, Oldenburg, Germany; Human Genetics, School VI—School of Medicine and Health Sciences, Carl von Ossietzky Universität Oldenburg, Oldenburg, Germany; Cologne Center for Genomics (CCG) and Center for Molecular Medicine Cologne (CMMC), University of Cologne, Cologne, Germany; Department of Ophthalmology, Justus-Liebig-University Giessen, Universitaetsklinikum Giessen and Marburg UKGM GmbH Giessen Campus, Giessen, Germany; Human Genetics, School VI—School of Medicine and Health Sciences, Carl von Ossietzky Universität Oldenburg, Oldenburg, Germany; Cologne Center for Genomics (CCG) and Center for Molecular Medicine Cologne (CMMC), University of Cologne, Cologne, Germany; Ulverscroft Eye Unit, School of Psychology and Vision Sciences, University of Leicester, Leicester LE2 7LX, UK; Department of Ophthalmology, University Hospitals of Leicester NHS Trust, Leicester, LE1 5WW, UK; Cologne Center for Genomics (CCG) and Center for Molecular Medicine Cologne (CMMC), University of Cologne, Cologne, Germany; Department of Ophthalmology, Justus-Liebig-University Giessen, Universitaetsklinikum Giessen and Marburg UKGM GmbH Giessen Campus, Giessen, Germany; Rare Retinal Disease Center, MVZ Augenärztliches Diagnostik- und Therapiecentrum Siegburg GmbH, Siegburg, Germany; RetinaScience, Bonn, Germany; Division of Human Medicine, School VI—School of Medicine and Health Sciences, Research Group Anatomy, Carl von Ossietzky Universität Oldenburg, Oldenburg, Germany; Research Center for Neurosensory Science, Carl von Ossietzky University Oldenburg, Oldenburg, Germany; Department of Neuropediatrics, University Children’s Hospital, Klinikum Oldenburg, Oldenburg, Germany; Ulverscroft Eye Unit, School of Psychology and Vision Sciences, University of Leicester, Leicester LE2 7LX, UK; Human Genetics, School VI—School of Medicine and Health Sciences, Carl von Ossietzky Universität Oldenburg, Oldenburg, Germany; Research Center for Neurosensory Science, Carl von Ossietzky University Oldenburg, Oldenburg, Germany

**Keywords:** idiopathic congenital nystagmus, idiopathic infantile nystagmus (IIN), voltage-gated Ca^2+^ channel (VGCC), high-voltage activated calcium channel, Ca_V_β subunit

## Abstract

Infantile nystagmus (IN) is a common neuro-ophthalmological disorder that presents as early-onset involuntary oscillations of the eyes. Here, we report a novel genotype-phenotype correlation that associates sequence alterations in the calcium voltage-gated channel auxiliary subunit beta 3 (*CACNB3*) gene, encoding the Ca_V_β3 protein, with idiopathic infantile nystagmus (IIN). Linkage analysis, whole exome and Sanger sequencing identified a homozygous missense mutation (c.316G>C) in *CACNB3* co-segregating with IIN. Our calcium imaging experiments suggest that the p.Gly106Arg mutation in the Src homology 3 domain of Ca_V_β3 may impair voltage-gated calcium channel function at the plasma membrane and may increase ligand-triggered inositol trisphosphate receptor mediated calcium release at the endoplasmic reticulum. Co-localization studies indicate reduced plasma membrane localization of the calcium channel. We propose *CACNB3* to be a novel gene associated with IIN. Our findings point towards an important role of calcium-signalling in IIN and may contribute to deciphering its aetiology.

## Teaser

Jüschke *et al.* report a novel genotype-phenotype correlation between a *CACNB3* (Ca_V_β3) mutation and idiopathic infantile nystagmus. The mutation impairs voltage-gated calcium channel function at the plasma membrane and increases calcium release from the endoplasmic reticulum. This discovery highlights the role of calcium signalling in the aetiology of nystagmus.

## Graphical Abstract

**Graphical Abstract:**
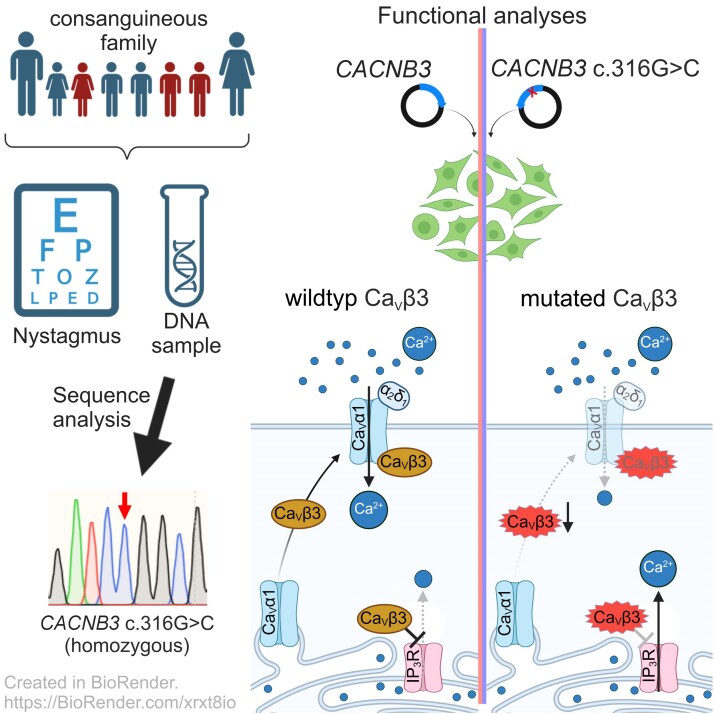

## Introduction

Nystagmus presents as ocular oscillation characterized by an involuntary, periodic movement in which one or both eyes drift away from the point of fixation. This so-called slow phase is followed by either a quick, re-fixating jerk or a slower, pendular movement to resume fixation.^1,2^ In contrast to acquired forms of nystagmus which appear later in life, infantile nystagmus (IN) usually appears within the first six months after birth. It can either be idiopathic or associated with retinal or neurological aetiologies.^2^ With a reported prevalence of about 2–3 in 10 000 in the European population, idiopathic infantile nystagmus (IIN) or idiopathic congenital nystagmus is among the most common types of IN.^3,4^ IIN may be associated with reduced visual acuity, strabismus, astigmatism, and abnormal head posture.^5^

Over the last years, an increasing number of genes has been associated with IN, the most frequently mutated IN gene being *FRMD7* (FERM Domain Containing 7) on chromosome Xq26.2.^6-8^ Interestingly, two genes encoding Ca_V_α1 subunits of voltage-gated calcium channels (VGCC), *CACNA1A*^9,10^ and *CACNA1F,*^11,12^ have previously been associated with nystagmus pointing towards a role of calcium signalling in its aetiology.

VGCCs transduce changes of membrane potential into changes of cytoplasmic calcium ion (Ca^2+^) levels thereby regulating a plethora of physiological processes including neurotransmission, gene expression, and signalling pathways. VGCCs are composed of three major subunits, a pore forming Ca_V_α1 subunit and two auxiliary subunits, Ca_V_α2δ and Ca_V_β.^13-15^ Four different types of Ca_V_β proteins are encoded by the genes *CACNB1* to *CACNB4*, each occurring with multiple splice variants. The corresponding proteins contain two highly conserved domains, a Src homology 3 (SH3) domain and a guanylate kinase (GK) domain, separated by a HOOK region and flanked by variable N- and C-termini.^16,17^

The calcium voltage-gated channel auxiliary subunit beta 3 (*CACNB3*) gene is located on chromosome 12q13.12 and encodes the auxiliary Ca_V_β3 subunit that was shown to regulate the properties of VGCCs of the subtypes Ca_V_1.2 (L-type) and Ca_V_2.2 (N-type).^18-21^ To date, three major functions were associated with Ca_V_β3: (i) Ca_V_β3 accelerates the influx of Ca^2+^ through VGCCs and changes opening and closing kinetics of the channel at the plasma membrane.^16^ (ii) Ca_V_β3 facilitates trafficking of the VGCC pore forming Ca_V_α1 subunit to the plasma membrane.^22-24^ (iii) Ca_V_β3 regulates the release of intracellular Ca^2+^ from the endoplasmic reticulum (ER) independently from its VGCC-related functions.^25,26^

We identified a homozygous missense alteration in *CACNB3* co-segregating with IIN. Using Ca^2+^ live-cell imaging and co-localization analysis, we found that the sequence change in Ca_V_β3 compromises its function on several levels. *CACNB3* thus is a novel gene associated with IIN.

## Materials and methods

### Patients and ethics statement

The study was conducted according to the Declaration of Helsinki and was approved by the local ethics committees [Hannover Medical School (MHH) ethics committee: MHH 2576-2015; Medizinische Ethikkommission, University of Oldenburg: 2018-097].

**Figure 1:**
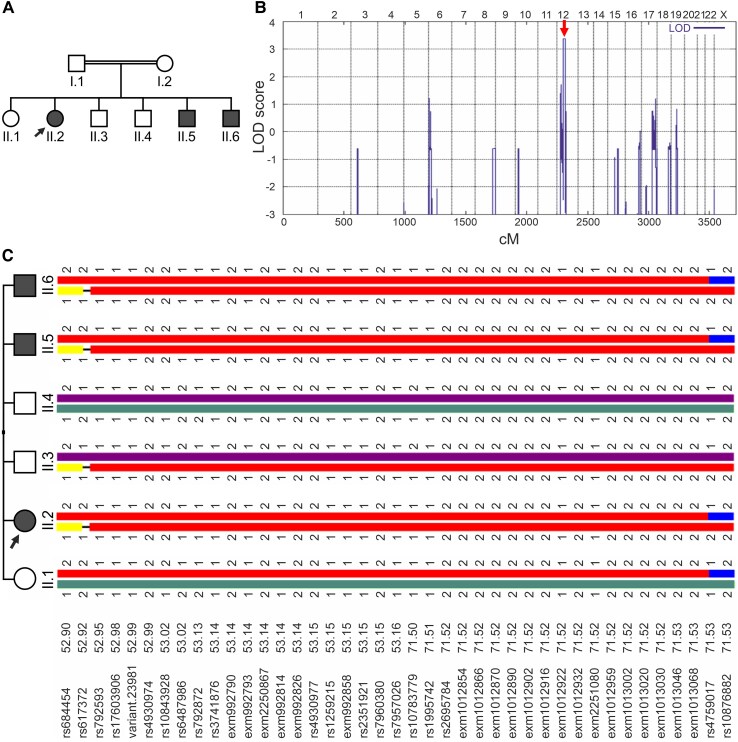
Genetic analysis of the family affected by IIN. (A) Pedigree of the family with three affected members (II.2, II.5, II.6) suffering from IIN (filled symbols). The index patient (II.2) is marked by an arrow. Circles represent females, squares represent males. (B) Parametric linkage analysis of the family was performed with 24 209 selected single nucleotide polymorphism (SNP) markers from the Illumina HumanCoreExome-12v1-1 BeadChip and revealed a significant LOD score on chromosome 12 (indicated by a red arrow). (C) Haplotype reconstruction for the linkage region on chromosome 12 of all six children of the family included in the study. See also Supplementary Fig. 1 for the haplotype reconstruction of all eight family members. cM: centi Morgan; LOD: logarithm of the odds.

Eight members of a Lebanese family, three females and five males, were recruited for the study, three of which were diagnosed with IIN. The pedigree (Fig. 1A) was assembled based on clinical examinations, interviews, and/or ophthalmological reports. Clinical examination and ophthalmic evaluation included the following measurements: Ocular biomicroscopy, fundus autofluorescence (FAF), and optical coherence tomography (OCT). In addition to DNA samples from the family, genomic DNA from 63 additional IIN cases and 48 controls were investigated.

We were not able to collect additional IIN patients with *CACNB3* variants through the GeneMatcher platform.^27^

### Genetic analyses

#### DNA isolation

Genomic DNA was extracted from peripheral blood samples of three affected and five unaffected family members (Fig. 1) using the MagCore nucleic acid extraction kit (Labgene Scientific SA, Châtel-Saint-Denis, Switzerland) according to manufacturer’s instructions. For DNA extraction from patients with IIN, the Maxwell® 16 Cell DNA Purification Kit (Promega) with the Maxwell RSC robot (Promega) was used. DNA from unaffected controls was isolated using the Puregene Blood Core Kit B (QIAGEN GmbH, Hilden, Germany).

#### Whole-exome sequencing

Whole-exome sequencing (WES) of genomic DNA extracted from blood of the nystagmus-index patient was performed at the Cologne Center for Genomics, University of Cologne (http://ccg.uni-koeln.de) as described previously.^28^ Briefly, the SeqCap EZ Human Exome Library v2.0 (Roche NimbleGen Inc., Madison, WI) was used to enrich for exonic and adjacent splice site sequences, and analysis was performed with the paired-end 2× 100 bp protocol and v3 chemistry on the Illumina HiSeq 2000 system. After read mapping to the human reference genome (GRCh37/hg19) using the BWA-SW alignment algorithm, Varbank (https://varbank.ccg.uni-koeln.de/) was used for variant calling. Filtering of high-quality variant calls was performed using the following parameters: variants occurring with a frequency of ≤0.02 compared with the 1000 genomes database, minor allele frequency (MAF) ≤0.02, SIFT ≤0.05, PolyPhen2 ≤0.85, variants depicted as ‘disease-causing’ according to mutationtaster,^29^ appropriate sequence coverage and quality. As references, the sequences NM_000725.3 for *CACNB3*, NM_001098531.2 for *RAPGEF3*, NM_003482.3 for *KMT2D*, and NM_000424.3 for *KRT5* were used. WES data were visualized using Integrative Genomics Viewer.^30,31^

#### Linkage analysis

Linkage analysis was performed at the Cologne Center for Genomics as described previously.^32^ Briefly, extracted DNA from patients was analysed using Illumina HumanCoreExome-12v1-1 BeadChip (Illumina Inc., San Diego, CA) according to the manufacturer’s protocol. Linkage analysis was performed assuming autosomal recessive inheritance, full penetrance, consanguinity, and a disease gene frequency of 0.0001. Multipoint logarithm of the odds (LOD) scores were calculated using the program ALLEGRO.^33^ Haplotypes were reconstructed with ALLEGRO and presented graphically with HaploPainter.^34^

#### Sanger sequencing

Primers for amplification and sequencing of *CACNB3* (NM_000725.3) and *RAPGEF3* (NM_006105.5) are available upon request. Sanger sequencing was performed using the 3130XL genetic analyser and the BigDye® Terminator v3.1 Cycle Sequencing Kit (Applied Biosystem, Carlsbad, CA, USA). Sequences were analysed with the SeqScape software v2.5 (Applied Biosystem) and the SnapGene software (GLS Biotech, Boston, MA, USA). In addition to DNA samples from the family, genomic DNA from 63 additional IIN cases, lacking any known genetic cause in known IN-related genes, were analysed and compared with 48 controls. All exons and flanking introns of the *RAPGEF3* or *CACNB3* genes were analysed. These analyses did not reveal any homozygous or compound heterozygous alterations in *RAPGEF3* or *CACNB3* (Supplementary Table 1).

### Multiple sequence alignment

Protein sequences of Ca_V_β3 were aligned using Clustal Omega^35^ (version 1.2.4): human: *Homo sapiens* NP_000716.2; rat: *Rattus norvegicus* NP_036960.1; mouse: *Mus musculus* NP_031607.2; frog: *Xenopus laevis* NP_001079266.1; chicken: *Gallus gallus* XP_025001452.1; fish: *Danio rerio* XP_005162052.1; fly: *Drosophila melanogaster* NP_523546.1; nematode: *Caenorhabditis elegans* NP_491193.2; and protein sequences of human Ca_V_β paralogs: Ca_V_β1: NP_000714.3, Ca_V_β2: NP_000715.2, and Ca_V_β4: NP_000717.2.

### Cell culture, plasmids, and transfection

Human embryonic kidney 293T (HEK293T) cells were cultured in Dulbecco's Modified Eagle’s Medium (DMEM) supplemented with 10% foetal bovine serum, 1% L-glutamine and 1% penicillin–streptomycin under standard cell culture conditions (37°C, 5% CO_2_). The day before transfection, approximately 300 000 cells were seeded on poly-L-lysine (PLL; Sigma-Aldrich) coated coverslips. For calcium imaging, plasmids encoding different VGCC subunits^36^ and tdTOMATO were mixed in equimolar amounts to a total of 2 µg in DMEM and transfected using polyethylenimine (PEI, 1 mg/ml, Sigma-Aldrich) in a 1:3 ratio. For co-localization analyses, plasmids expressing *CACNB3* (wild-type or mutated), *GFP-CACNA1B*, and *mCherry-CD9-10* were mixed in equimolar amounts to a total of 2 µg in DMEM and transfected using PEI. All plasmids used in this study are listed in Supplementary Table 2. The c.316G>C mutation in *CACNB3* was generated using standard mutagenesis methods and verified by Sanger sequencing.

### RNA isolation, cDNA synthesis, and RT-PCR

RNA isolation was performed using the NucleoSpin® Mini Kit for RNA purification (Macherey-Nagel, Düren, Germany) according to the manufacturer’s instructions. SuperScript III Reverse Transcriptase (Thermo Fisher Scientific) with random primers was used for cDNA synthesis from 1 µg of total RNA following the manufacturer’s instructions. Primers used for RT-PCR are listed in Supplementary Table 3.

### Calcium live cell imaging

HEK293T cells were cultured on PLL coated coverslips. One day after transfection, cells were loaded with 2 µM Fura 2-AM (Thermo Fisher Scientific) in HEPES buffer (137 mM NaCl, 5 mM KCl, 5.6 mM D-glucose, 20 mM HEPES, 0.59 mM KH_2_PO_4_, 0.56 mM Na_2_HPO_4_, 1.4 mM CaCl_2_, 0.9 mM MgSO_4_, 10 mM NaHCO_3_, pH 7.4) for 30 min at 37°C, 5% CO_2_ in the dark. Cells were secured in a perfusion chamber mounted onto an inverted microscope equipped with a calcium imaging system (Leica), and all solutions were applied by bath perfusion (1 ml/min).

For measuring calcium influx via VGCCs, cells were maintained in Krebs-Ringer’s solution (119 mM NaCl, 2.5 mM KCl, 1 mM NaH_2_PO_4_, 2.5 mM CaCl_2_, 1.3 mM MgCl_2_, 20 mM HEPES, 11 mM D-glucose, pH 7.4) and stimulated using 55 mM KCl in Krebs-Ringer’s solution, followed by 10 µM ATP in Krebs-Ringer’s solution to confirm cell responsiveness at the end of the experiment.

For measuring calcium release from intracellular stores, cells were maintained in Tyrode’s solution (140 mM NaCl, 4 mM KCl, 2 mM MgCl_2_, 10 mM HEPES, 10 mM D-glucose, pH 7.4) and stimulated using 10 µM ATP in Tyrode’s solution.

Every 2 s, emission at 510 nm was recorded upon excitation at 340 and 380 nm using a Leica DMi8 microscope equipped with a X-Cite 200 DC illuminator (Excelitas Technologies), a HC PL FLUOTAR 20x/0.80 Oil objective (Leica Microsystem), and a cooled Leica DFC9000 GT Camera. Individual cells were traced and [Ca^2+^] kinetics (F340/F380 nm) recorded. Raw data and images were analysed with the Leica application Suite X 3.4.2.18368 (2018) software. After background correction, the ratio of the 340 and 380 nm channel was calculated.

### Colocalization analysis

HEK293T cells were seeded on PLL coated coverslips in 12-well plates 24 h after transfection with plasmids expressing *CACNB3* (wild-type or mutated), *GFP-CACNA1B*, and *mCherry-CD9-10*. On the next day, cells were fixed in 4% paraformaldehyde for 10 min, washed three times with phosphate-buffered saline containing 0.05% Tween-20, and mounted with mounting medium containing 4′,6-diamidin-2-phenylindol (DAPI) (Fluoromount-G, SouthernBiotech). Images were obtained using a Zeiss Axio Observer 7 microscope (Carl Zeiss, Oberkochen, Germany) with a 40x/0.6 Korr Ph2 M27 plan apochromat objective. Images were processed using ZEN Blue software version 2.3 SP1 (Carl Zeiss). Colocalization evaluation was performed blinded to the transfected plasmids by counting the number of cells showing and not showing colocalization of GFP- and mCherry-signal at the plasma membrane.

### Statistical analysis

All data and statistical analyses were performed in R (version 4.4.3) using the lme4 (1.1-37), lmerTest (3.1–3), and emmeans (1.11.2-00002) packages. For calcium live cell imaging experiments, imaging traces from individual cells marked as region of interest were used for feature extraction (resting calcium level, peak amplitude, and area under the curve). Linear mixed effects models (LMM) were fitted by restricted maximum likelihood (REML), with condition (mock, wild-type, mutation) as fixed effect and experimental replicate as random intercept to account for batch-to-batch variability and non-independence of cell measurements within each experiment. Statistical significances were assessed and estimated marginal means (EMM) were compared pairwise using the emmeans package with Tukey’s adjustment for multiple comparisons. The number of independent experiments and total cell counts per condition (mock, wild-type, mutation) are provided in the corresponding figure legends.

A generalized linear mixed model (GLMM) with a binomial error distribution and logit link function was employed to analyse the co-localization experiments (binary response variable co-localization). The model included condition (wild-type, mutation) as fixed effect and experimental replicate as random intercept to account for repeated measures and variability across replicates. *Post hoc* comparison of EMM was performed using the emmeans package. *P* values < 0.05 were considered to indicate statistical significance: *P* ≥ 0.05, not significant (n.s.); *P* < 0.05, *; *P* < 0.01, **; *P* < 0.001, ***. Data are presented as EMM ± standard error (SE).

## Results

### Clinical findings

A two-generation Lebanese family of eight members including three patients (II.2, II.5, II.6) was diagnosed with familial IIN. The family history, as reported by the parents, suggested consanguinity and an autosomal recessive mode of inheritance (Fig. 1A). The female index patient II.2 and her two brothers II.5 and II.6 were affected by IIN and refractive errors including astigmatism since early childhood.

The female index patient II.2 showed high intensity nystagmus which increased on fixation. This patient had a discrete and alternating head tilt (5°–10°). In addition, strabismus sursoadductorius with dissociated vertical deviation and minor conjunctiva scars on the right eye were noted. The macula, retina, and optic nerve appeared to be healthy. Magnetic resonance imaging of the head was unremarkable. Cognitive abilities, blood glucose, and skin pigmentation and elasticity were normal.

The two brothers, II.5 and II.6, were affected by nystagmus with head tilt to the left. The nystagmus intensity was reduced with right gaze suggestive of an eccentric null point. Both were not affected by additional eye diseases. Moreover, the index patient II.2 and the brother II.5 were diagnosed with a slight tendency for head-nodding. Diagnostic genetic testing of the index patient II.2 excluded variants in the coding region and exon-intron borders of known IN genes.

The three affected siblings of the family were examined clinically at different ages (8–21 years of age). The nystagmus partly inhibited detailed ocular examination. In all of them, biomicroscopic examination of the anterior and posterior segments of both eyes was unremarkable. FAF indicated normal distribution of lipofuscin in all eyes. Due to nystagmus, volume-scan OCT was not possible. Multiple single-scan OCTs revealed normal foveal and macular configuration of the retinal layers in all eyes. In summary, visual fields, ophthalmoscopy and retinal imaging were normal and did not reveal retinal disorders as a possible cause for the nystagmus.

A difference between the three siblings was noted regarding refractive error and visual acuity. Patient II.2 had a moderate hyperopia and astigmatism (RE +3.50 sph −2.00 cyl 145 axis = 0.2; LE +4.50 sph −2.75 cyl 14 axis = 0.2). Her 10-year-old brother II.5 presented with a mild myopia and astigmatism (RE −0.75 sph −1.25 cyl 168 axis = 0.4; LE −0.50 sph −1.75 cyl 10 axis = 0.4), whereas her 8-year-old brother II.6 presented with a moderate hyperopia and astigmatism (RE +2.25 sph −2.75 cyl 17 axis = 0.4; LE +3.00 sph −2.75 cyl 11 axis = 0.3).

### Genetic analyses

To identify the genetic cause of IIN in the affected family members, WES was performed for the index patient II.2. Linkage analysis revealed a linkage interval on chromosome 12 with a significant LOD score of 3.38 between the positions 31409579 and 56691600 (NCBI build 37, Fig. 1B and C, Supplementary Fig. 1). In this region, four non-synonymous homozygous variants were detected in different genes (Table 1). All four variants co-segregated within the family, while three variants were considered rare enough to be associated with the diagnosis (<0.1% according to gnomAD, https://gnomad.broadinstitute.org) (Table 1).

**Table 1:** Homozygous co-segregating variants within the linkage interval

Gene	Exon	NT change	Consequence	Reference seq	gnomAD
*CACNB3*	4	c.316G>C	p.G106R	NM_00725.3	0.000002480
*RAPGEF3*	15	c.1546C>T	p.R516*	NM_001098531.2	0.000004775
*KMT2D*	31	c.7670C>T	p.P2557L	NM_003482.3	0.007608
*KRT5*	2	c.715C>T	p.R239C	NM_000424.3	0.00004151

Both parents (I.1, I.2) and two of the three unaffected children (II.1, II.3) comprise the variants in a heterozygous state while all variants are present in a homozygous state in the three affected children (II.2, II.5, II.6): a nonsense alteration in *RAPGEF3* and three missense alterations in *CACNB3*, *KRT5* and *KMT2D* (Table 1). Using Sanger sequencing, the variants were confirmed to co-segregate with the disease and within the family (for *CACNB3* see Supplementary Fig. 2).

The variants in the genes *KMT2D* (*Lysine Methyltransferase 2D*) and *KRT5* (*Keratin 5*) were considered unlikely to be causative mutations for IIN, since they are known to be associated with Kabuki syndrome^37^ or skin diseases like Dowling-Degos disease and Epidermolysis bullosa,^38,39^ respectively. Additionally, the *KMT2D* alteration was too frequent to be considered further as a nystagmus associated gene alteration.

**Figure 2:**
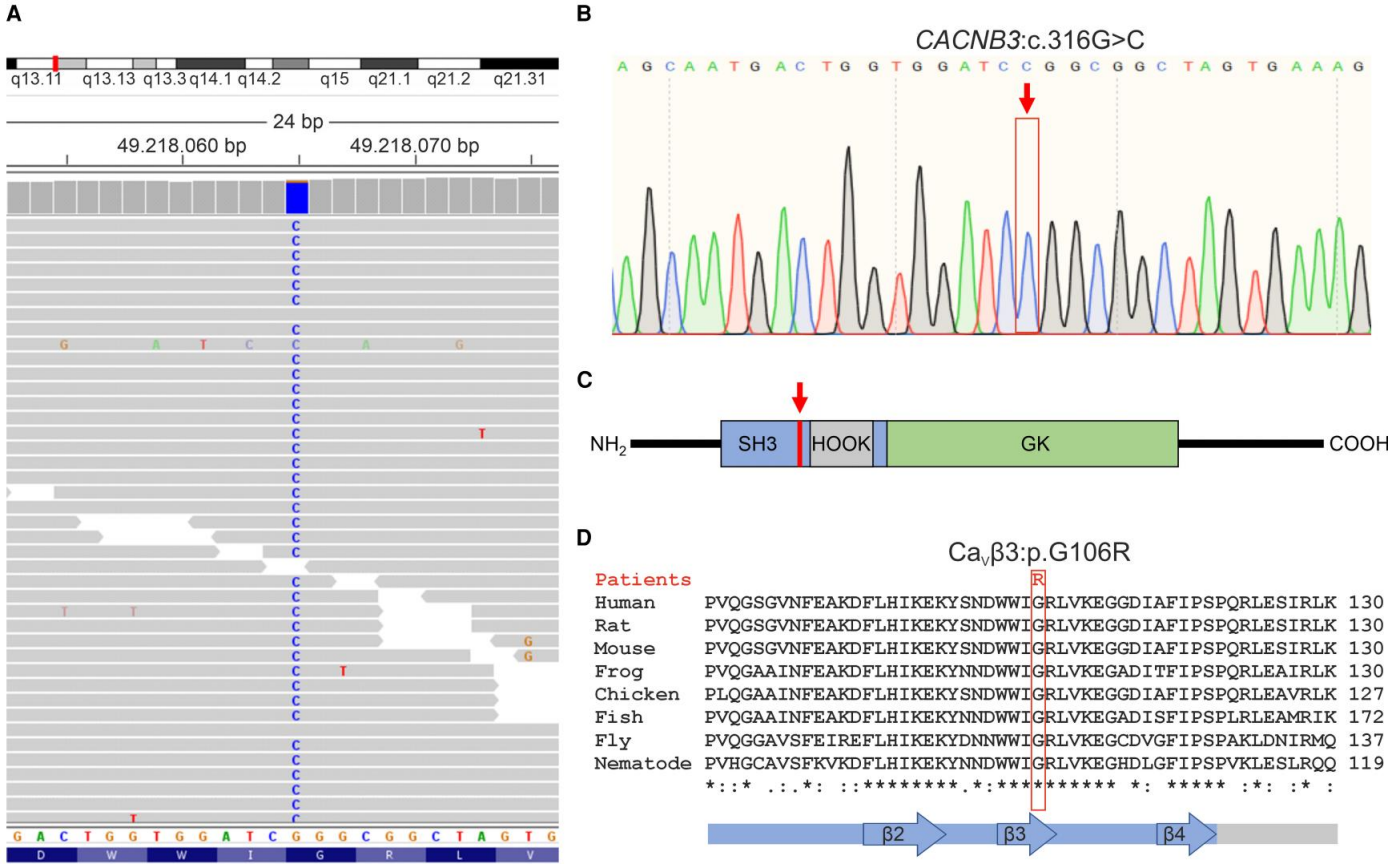
Sequence analyses identified a *CACNB3*:c.316G>C mutation. (A) WES analysis showed a homozygous *CACNB3*:c.316G>C mutation (highlighted in blue). (B) Sanger sequencing confirmed the homozygous *CACNB3*:c.316G>C mutation (marked by a red arrow) in the index patient II.2 (and the other affected family members, see also Supplementary Fig. 2). (C) Scheme of the protein domain structure of the *CACNB3* gene product, Ca_V_β3. The SH3 domain is shown in blue, the HOOK region in grey, the GK domain in green. The position of the p.G106R missense mutation in Ca_V_β3 within the SH3 domain is highlighted in red by an arrow. (D) Multiple sequence alignment of Ca_V_β3. Secondary structure elements are shown below the alignment (coloured like in panel C). The mutation c.316G>C causes an exchange of a highly conserved glycine (G) to an arginine (R, red) in the third β-sheet (β3) of the SH3 domain of Ca_V_β3. See also Supplementary Fig. 3 for an alignment of all human Ca_V_β paralogs. Supplementary Fig. 4 shows the variant allele frequencies along the Ca_V_β3 sequence. bp: base pairs; * (asterisk)—single, fully conserved residue; : (colon)—conservation between groups of strongly similar properties; . (period)—conservation between groups of weakly similar properties.

In the subsequent analyses, we verified the functional impact of the *CACNB3* missense alteration. Following reasons supported our decision: (i) The homozygous c.316G>C substitution in exon 4 of *CACNB3* (Fig. 2A and B) causes an exchange of a highly conserved glycine (G) to an arginine (R) in the third β-sheet (β3) of the Src homology 3 (SH3) domain in Ca_V_β3 (Fig. 2C and D). The SH3 domain of Ca_V_β3, and especially the proximity of glycine 106, is evolutionary conserved (Fig. 2D) and conserved in all human Ca_V_β paralogs Ca_V_β1–4 (Supplementary Fig. 3), thus strongly supporting its functional relevance. In the coding region of *CACNB3*, variant allele frequencies are significantly lower at the SH3- and GK-domain compared with the remaining part of the protein (Supplementary Fig. 4). Furthermore, no homozygous nonsense variant and only 15 different loci carrying homozygous non-synonymous variants (in 53 individuals) were reported (gnomAD v4.1.0, see Supplementary Fig. 5). (ii) In contrast, single knock-out mice of *RAPGEF3* (also known as *EPAC1*) or *RAPGEF4* (also known as *EPAC2*) showed no overt physiological abnormalities while double knock-outs exhibited severe deficits, strongly indicating functional redundancy of EPAC proteins in the brain *in vivo*.^40^ (iii) The eyes of *RAPGEF3* (*EPAC1*) knock-out mice did not show any phenotype in retinal histology or retinal neuronal function.^41^ (iv) *CACNB3* knock-out mice were reported to show delayed axonal refinement in the visual pathway^42^ reminiscent to down regulation of the nystagmus gene *FRMD7* which has been shown to alter neurite outgrowth *in vitro*.^43^ Thus, *CACNB3* is considered our best candidate to be associated with the IIN described in the three patients characterized herein.

### Functional analyses

**Figure 3:**
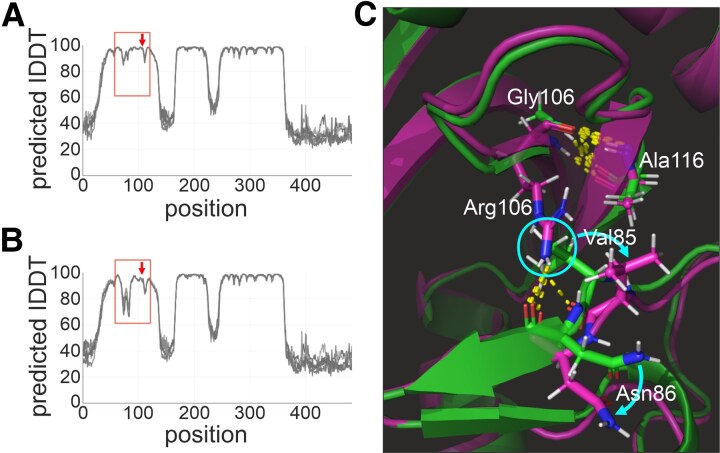
Structural modelling of Ca_V_β3 p.G106R and wild-type Ca_V_β3 using Alphafold2. (A) Predicted local Distance Difference Test (lDDT) for wild-type Ca_V_β3. (B) Predicted lDDT for Ca_V_β3 p.G106R. The red box highlights the region mostly affected by the mutation. The red arrow points to the amino acid position 106. The five overlaid curves represent the top 5 predictions (rank 1 to 5) of Alphafold2. (C) Overlay of the ribbon diagrams of the predicted 3D models of wild-type Ca_V_β3 (green) and Ca_V_β3 p.G106R (pink). The sidechain of Arg106 (pink, compared with Gly106 in green) is displacing the sidechains of Val85 (see cyan circle) and Asn86 from their native orientations (shown in green) to adopt distorted conformations (shown in pink). The directions of the displacements are indicated by cyan arrows. The peptide backbone H-bonds to Ala116 are maintained by p.G106R. H-bonds are shown as yellow dots.

In order to evaluate the structural consequences of the p.G106R mutation, we performed 3D structure predictions comparing wild-type and mutated Ca_V_β3 using Alphafold2.^44,45^ The predicted local Distance Difference Test (lDDT) indicates that the structural distortions of the missense alteration are strongest not at the site of amino acid position 106 but about 20 amino acids upstream (Fig. 3A and B). Comparing the 3D structures, both, Gly106 (wild-type) and Arg106 (mutant) maintain peptide backbone H-bonds with Ala116 (Fig. 3C). In the mutated Ca_V_β3, however, the sidechain of Arg106 is displacing the sidechains of Val85 and Asn86 from their native orientations (Fig. 3C). This may affect binding interactions of the SH3 domain of Ca_V_β3.

Since Ca_V_β3 can affect intracellular Ca^2+^ levels both, by changing VGCC properties at the plasma membrane and by regulating Ca^2+^ release from the ER, we employed the ratiometric Ca^2+^ indicator Fura-2 as a consistent readout for characterizing the impact of the Ca_V_β3 p.G106R mutation on both of these functions.

To investigate the impact of the Ca_V_β3 p.G106R mutation on the VGCC properties, we co-expressed the VGCC subunits Ca_V_2.2 (*CACNA1B*) and Ca_V_α_2_δ_1_ (*CACNA2D1*) together with either empty vector (mock), wild-type or mutated Ca_V_β3. We confirmed comparable expression of the three transfected VGCC subunits by RT-PCR (Supplementary Fig. 6). Using live-cell Ca^2+^ imaging, we determined changes in intracellular [Ca^2+^] upon potassium-induced plasma membrane depolarization (in the presence of extracellular Ca^2+^).

**Figure 4:**
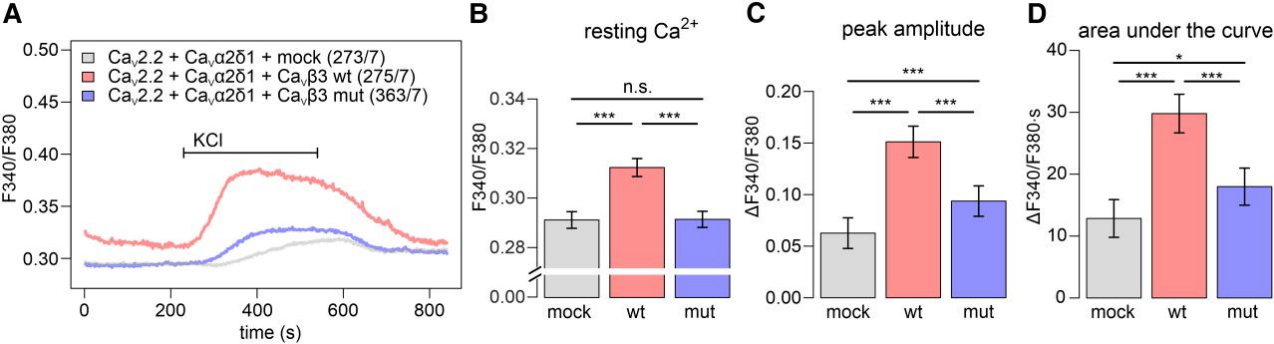
Influx of extracellular Ca^2+^ upon membrane depolarization is significantly reduced by expression of Ca_V_β3 p.G106R (Ca_V_β3 mut) as a VGCC subunit. (A) Averaged Fura-2 ratiometric traces before and after potassium-induced plasma membrane depolarization in HEK cells co-expressing either the wild-type Ca_V_β3 (wt, red), or the mutated Ca_V_β3 (mut, blue), or the empty vector (mock, grey) together with the VGCC subunits Ca_V_2.2 and Ca_V_α_2_δ_1_ in the presence of extracellular Ca^2+^. VGCC subunits were expressed at comparable levels (Supplementary Fig. 6). Only responsive cells showing area under the curve > 5 were used for the analysis. Number of measured cells (X) from independent experiments (Y) are indicated as (X/Y) in the legend. (B)–(D) Co-expression of mutated Ca_V_β3 as VGCC subunit leads to significantly reduced levels of resting Ca^2+^ in the cytoplasm (B), reduced Ca^2+^ peak amplitude (C), and reduced area under the curve (D). (B)–(D) Statistics: Resting Ca^2+^, peak amplitude, and area under the curve are presented as EMM ± SE from 536 (mock), 381 (wt), and 650 (mut) cells recorded in seven independent runs of the experiment. For each parameter, a LMM was fitted with condition (mock, wt, mut) as fixed effect and experimental replicate as random intercept (value ∼ condition + (1 | experiment)). Statistical significances were determined by *post hoc* comparisons among groups after model fitting (emmeans R package). See Supplementary Tables 4–9 for detailed information on the statistical test results. KCl: potassium chloride.

Our results show that the influx of extracellular Ca^2+^ through VGCCs upon membrane depolarization was significantly reduced by the sequence alteration Ca_V_β3 p.G106R (Fig. 4). Cells expressing mutated Ca_V_β3 seemed to respond slower and less efficient to depolarization (Fig. 4A). In addition, they exhibited significantly lower resting Ca^2+^ levels (Fig. 4A and B), indicating basal activity or Ca^2+^ leakage of overexpressed wild-type VGCCs. Likewise, peak amplitude and area under the curve were significantly reduced in cell expressing mutated Ca_V_β3 (Fig. 4A and C, D). However, Ca^2+^ influx was still higher than in cells lacking Ca_V_β3, indicating that the p.G106R mutation is a hypomorph, showing residual functional capacity to modulate VGCC properties (Fig. 4C and D).

**Figure 5:**
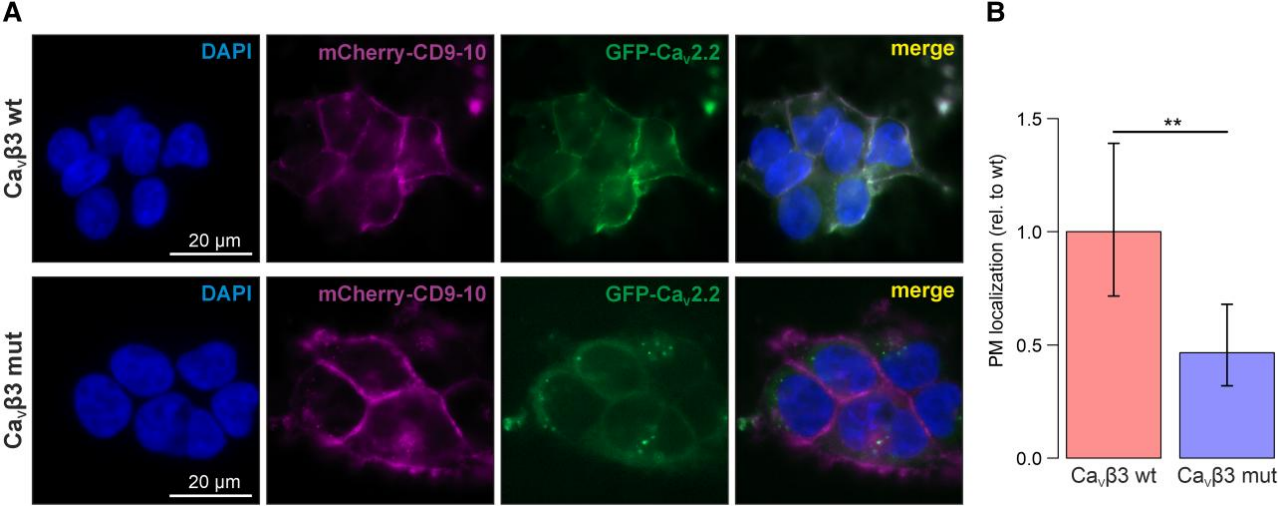
Plasma membrane localization of GFP-Ca_V_2.2 is reduced by expression of Ca_V_β3 p.G106R. (A) Exemplary images of HEK cells co-expressing either the wild-type Ca_V_β3 (Ca_V_β3 wt, top panels) or the mutated Ca_V_β3 (Ca_V_β3 mut, bottom panels) together with GFP-Ca_V_2.2 (green) and the plasma membrane marker mCherry-CD9-10 (magenta). Nuclei are counterstained with DAPI. Scale bar = 20 µm. (B) Quantification of co-localization of mCherry-CD9-10 and GFP-Ca_V_2.2 at the plasma membrane (PM) in Ca_V_β3 wt (red) and Ca_V_β3 mut (blue) co-expressing cells. Statistics: A GLMM was fitted with condition (wt, mut) as fixed effect and experimental replicate as random intercept (colocalization ∼ condition + (1 | replicate)). The EMM ± SE (from a total of 1882 cells analysed in eight independent replicates) are shown after transformation from logit scale to response scale and normalization to wild-type level. Statistical significance was determined by *post hoc* comparison after GLMM fitting (*P* = 0.0037). See Supplementary Tables 10 and 11 for detailed information on the statistical test results. GFP: green fluorescent protein.

To evaluate if Ca_V_β3 p.G106R affects trafficking of VGCCs to the plasma membrane, we co-expressed either wild-type or mutated Ca_V_β3 together with the GFP-tagged VGCC pore forming subunit GFP-Ca_V_2.2 (*GFP-CACNA1B*) and the plasma membrane marker mCherry-CD9-10 (Fig. 5). Co-localization analysis revealed a significant reduction of plasma membrane localization of GFP-Ca_V_2.2 upon Ca_V_β3 p.G106R co-expression compared with wild-type Ca_V_β3 (GLMM: *P* = 0.0037, Fig. 5B).

**Figure 6:**
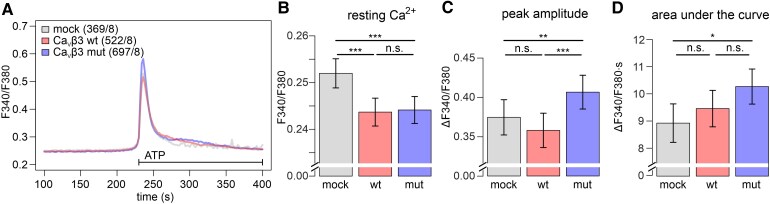
Mutated Ca_V_β3 fails to decrease ATP-induced release from intracellular Ca^2+^ stores. (A) Averaged Fura-2 ratiometric traces before and during ATP stimulation in HEK cells expressing wild-type Ca_V_β3 (wt, red), or mutated Ca_V_β3 (mut, blue), or empty vector (mock, grey) in the absence of extracellular Ca^2+^. Wild-type and mutated Ca_V_β3 were expressed at comparable levels (Supplementary Fig. 7). Number of measured cells (X) from independent experiments (Y) are indicated as (X/Y) in the legend. (B) Expression of mutated Ca_V_β3 does not significantly affect resting Ca^2+^ compared with wild-type Ca_V_β3. (C) In contrast to wild-type Ca_V_β3, mutated Ca_V_β3 increases Ca^2+^ peak amplitude upon ATP-stimulation. (D) Area under the curve is not significantly altered by mutated Ca_V_β3 expression compared with wild-type Ca_V_β3. (B)-(D) Statistics: Resting Ca^2+^, peak amplitude, and area under the curve are presented as EMM ± SE from 369 (mock), 522 (wt), and 697 (mut) cells recorded in eight independent runs of the experiment. For each parameter, a LMM was fitted with condition (mock, wt, mut) as fixed effect and experimental replicate as random intercept (value ∼ condition + (1 | experiment)). Statistical significances were determined by *post hoc* comparisons among groups after model fitting (emmeans R package). See Supplementary Tables 12–17 for detailed information on the statistical test results.

Independent from its function as a VGCC subunit, Ca_V_β3 has been shown to regulate cytosolic Ca^2+^ levels through binding to the inositol trisphosphate (IP_3_) receptor (IP_3_R) and thereby inhibiting the release of stored Ca^2+^ from the ER.^26^ This interaction is specific for the Ca_V_β3 isoform and is mediated by its SH3 domain.^25,26^ Opening of IP_3_ receptors and Ca^2+^ release from the ER is stimulated by binding of ATP to P2Y purinergic receptors.^46^ To analyse the consequences of the Ca_V_β3 p.G106R mutation in this process, we expressed either wild-type or mutated Ca_V_β3 in HEK cells and measured the ATP-evoked release of stored Ca^2+^ using Fura-2 in the absence of extracellular Ca^2+^. Similar expression of wild-type and mutant Ca_V_β3 was confirmed by RT-PCR (Supplementary Fig. 7). Compared with cells lacking Ca_V_β3 (mock), we found resting cytoplasmic Ca^2+^ levels to be slightly but significantly reduced by wild-type and mutated Ca_V_β3 expression (Fig. 6A and B). The ATP-induced Ca^2+^ release was significantly increased in cells expressing mutated Ca_V_β3 compared with both, cells lacking and cells expressing wild-type Ca_V_β3. This indicated that the p.G106R mutation abolished the regulation of Ca^2+^-release from the ER (Fig. 6C). Of note, the area under the curve did not differ significantly between wild-type and mutated Ca_V_β3 conditions, indicating that the Ca^2+^ storage capacity of the ER was not affected (Fig. 6D).

In summary, the Ca_V_β3 p.G106R mutation displayed two opposing effects on intracellular Ca^2+^ concentration: At the VGCC, it led to a reduced Ca^2+^ influx upon membrane depolarization, while at the IP_3_R, it increased Ca^2+^ release upon P2Y-mediated phospholipase C (PLC) activation and IP_3_ generation.

## Discussion

Congenital idiopathic nystagmus has so far been linked to only a few gene mutations with unclear aetiology. This study shows for the first time an association of congenital nystagmus with a homozygous missense mutation in *CACNB3* (NM_00725.3:c.316G>C) that co-segregates within a Lebanese family. The mutation is located in the highly conserved SH3 domain of Ca_V_β3 (Ca_V_β3 p.G106R), with the glycine residue being highly conserved among species from mammals to insects and worms. Nevertheless, the missense mutation may still maintain some residual activity. Ca_V_β3 exerts a variety of functions, including the regulation of intracellular Ca^2+^ levels and VGCC activities, which are important for development and neurotransmission. To assess the function of Ca_V_β3 both, at the ER and plasma membrane, we performed Ca^2+^ live cell imaging. Herein, we show that the Ca_V_β3 p.G106R mutation, identified in patients with congenital nystagmus, affects at least two major roles of Ca_V_β3: (i) the ability to increase Ca^2+^ influx through VGCCs and (ii) the regulation of Ca^2+^ release from the ER.

Expression of the mutated Ca_V_β3 led to aberrant VGCC function, as indicated by the disruption of membrane-depolarization induced Ca^2+^-influx. This may either be caused by altered interactions of Ca_V_β3 with Ca_V_α1 or compromised trafficking of Ca_V_α1 to the plasma membrane. Notably, a synergistic action of both of these Ca_V_β3 functions seems likely. While we were able to demonstrate very similar expression of both, wild-type and mutant *CACNB3* transcripts by RT-PCR, we cannot exclude the possibility that altered protein stability may indirectly contribute to the disease phenotype. Future studies will be required to investigate the electrophysiological and mechanistic consequences of the Ca_V_β3 p.G106R mutation on VGCC function.

Ca_V_β3 is primarily associated with Ca_V_2.2^18,19,47^ and Ca_V_1.2,^48^ and binds to the Ca_V_α1 subunit via a hydrophobic groove in its GK domain.^17,49^ In VGCC cryo-electron microscopy structures, G106 of Ca_V_β3 is not in close proximity to the α1-interacting domain (AID) of Ca_V_2.2 nor Ca_V_1.2.^47,48^ Hence, the physical interaction of Ca_V_β3 with the VGCC Ca_V_α1 subunit is probably not directly affected by the mutation. Our three-dimensional structure predictions rather indicate that the p.G106R mutation in the β3-sheet of the Ca_V_β3 SH3 domain may induce structural changes in the loop between the β1- and β2-sheets likely affecting SH3 domain properties und interactions. Nevertheless, we cannot completely exclude the possibility that the p.G106R mutation in the SH3 domain might influence the GK domain of Ca_V_β3 as well.

Interestingly, mutations in two other VGCC Ca_V_α1 subunits, *CACNA1A* and *CACNA1F*, have been associated with nystagmus.^9,10,12,50^ Of note, nystagmus is not the major phenotype associated with *CACNA1A* and *CACNA1F*: *CACNA1A* mutations cause a variety of neurological disorders including ataxia and hemiplegic migraines^51^ and *CACNA1F* mutations often were associated with incomplete congenital stationary night blindness.^52,53^ Although our clinical investigations (visual fields, ophthalmoscopy and retinal imaging) did not reveal retinal disorders, it cannot be completely excluded that a *CACNB3*-associated retinal origin of the nystagmus exists. Interestingly, the N-type VGCC blocker ziconotide may cause nystagmus as an adverse effect when applied as a painkiller in patients.^47,54^ The VGCC inhibitor gabapentin,^55^ on the other hand, has been successfully applied as IIN medication to reduce nystagmus intensity and improve visual acuity.^56^ Taken together, these data indicate that a proper functioning of VGCCs and Ca^2+^ homeostasis may play an important role in the aetiology of nystagmus.

Binding of ATP to P2Y receptors leads to PLC activation and cleavage of phosphatidyl inositol 4,5-bisphosphate (PIP_2_) into IP_3_ and diacylglycerol. Upon IP_3_ binding to the IP_3_R, Ca^2+^ is released from intracellular stores. Ca_V_β3 modulates this process by binding to the IP_3_R via its SH3 domain thereby decreasing the affinity of IP_3_R for IP_3_.^26^ This interaction has been shown to be a specific function of Ca_V_β3 that cannot be compensated by different Ca_V_β subtypes.^25^ As indicated by the increased Ca^2+^ peak amplitude, the p.G106R mutation is likely abolishing the inhibitory effect of Ca_V_β3. Structure predictions pointed towards Val85 and Asn86 as being severely displaced from their native conformations. It would be interesting to evaluate if these two amino acid residues are directly involved in the Ca_V_β3 SH3 domain binding to IP_3_R.

*CACNB3* knock-out mice exhibited apparently normal morphology but alterations in multiple components of neuronal Ca^2+^ channel current.^57,58^ Behaviourally, they showed reduced nociception, decreased anxiety, and increased aggression.^59,60^ In the visual pathway, knock-out mice displayed delayed axonal refinement^42^ reminiscent to down regulation of *FRMD7* which has been shown to alter neurite outgrowth *in vitro*.^43^ Interestingly, neurite outgrowth is promoted by calcium/calmodulin-dependent serine protein kinase (CASK) recruiting FRMD7 to the plasma membrane.^61^ An abnormal development of the afferent system has been associated with *FRMD7* mutations.^62^ Specifically, *FRMD7* mutant mice lack horizontally tuned direction-selective ganglion cells.^63^ Hence, a lack of regulation on the subcortical optokinetic pursuit system due to developmental defects may be a common cause of IN.^64^

We consider the homozygous nonsense mutation of *RAPGEF3* as being less likely associated with IIN based on the absence of any overt knock-out phenotype in mice.^40,41^ The RAPGEF3/EPAC1 protein exhibits high similarity to RAPGEF4/EPAC2 and is expressed in largely overlapping patterns throughout the brain.^65^ Individual knock-out mice for *RAPGEF3* or *RAPGEF4* displayed no detectable phenotypes, whereas double knock-outs showed severe deficits, highlighting the functional redundancy of EPAC in the brain *in vivo*.^40^ The absence of RAPGEF3/EPAC1 did also not induce any discernible phenotype in retinal histology or retinal neuronal function in mice.^41^ Nevertheless, we cannot completely exclude the possibility of digenetic effects of *CACNB3* together with *RAPGEF3*. The synergistic effect of CACNB3 and RAPGEF3 alterations may still be possible, as activation of EPAC was shown to facilitate the release of Ca^2+^ from the sarcoplasmic reticulum (SR) in a PLC and Ca^2+^/calmodulin kinase II (CaMKII) dependent manner^66-68^ and RAPGEF3/EPAC1 was described as a regulator of L-type calcium channels in a mouse model of atrial fibrillation and heart failure.^69^

Interestingly, Ca_V_β3 has been shown to regulate the activity of a specific isoform of the transcription factor Pax6.^70^ Pax6 is a master regulator of eye development and has been associated with nystagmus.^71,72^ It will be interesting to investigate whether Ca_V_β3 may act upstream of Pax6 and whether Ca_V_β3 mutations affect eye development and nystagmus via altered interaction with Pax6. Future studies will be necessary to distinguish in more detail the contribution of developmental and/or acute Ca^2+^-signalling defects in causing nystagmus.

In summary, we propose *CACNB3* to be a novel gene associated with IIN. Our results reveal an important role of Ca^2+^-signalling and/or homeostasis for the aetiology of nystagmus.

## Supplementary Material

fcag034_Supplementary_Data

## Data Availability

Raw data supporting the findings of this study are available from the corresponding author upon reasonable request. Custom written code generated for the analysis of the calcium live cell imaging data within this manuscript is publicly available online at https://github.com/Human-Genetics-Oldenburg.
