# Supplementary material for: CACNB3 defects are associated with infantile idiopathic nystagmus
Source: Brain Commun. 2026 Feb 7;8(2):fcag034. doi: 10.1093/braincomms/fcag034 (PMC12977960; doi:10.1093/braincomms/fcag034)
Supplement: fcag034_Supplementary_Data [file fcag034_supplementary_data.pdf]

Supplementary material

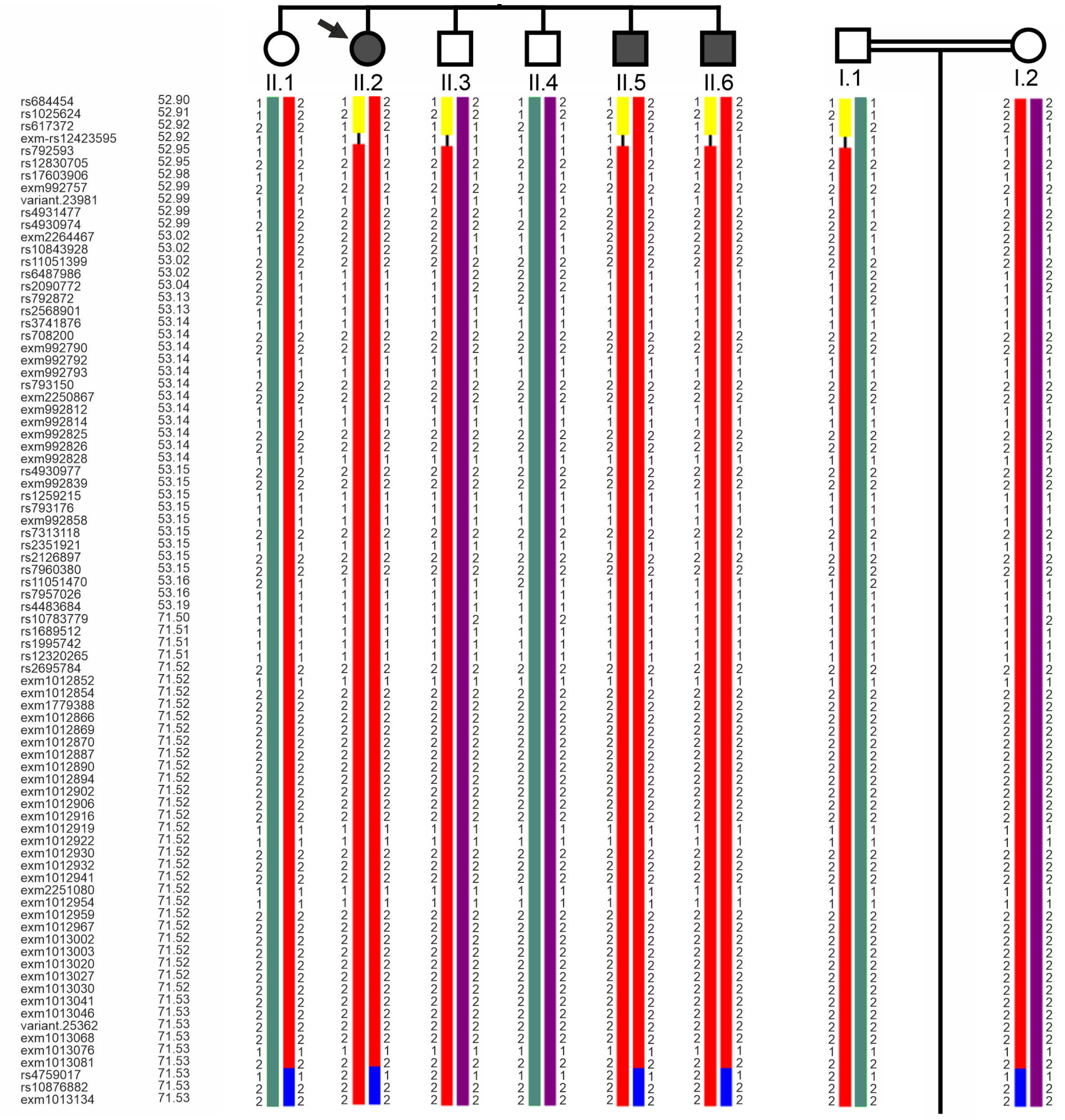

Supplementary Figure 1. Haplotype reconstruction for the linkage region on chromosome 12 of all eight family members included in the study. The pedigree of the family shows three affected members (II.2, II.5, II.6) suffering from IIN (filled symbols). The index patient (II.2) is marked by an arrow. Circles represent females, squares represent males.

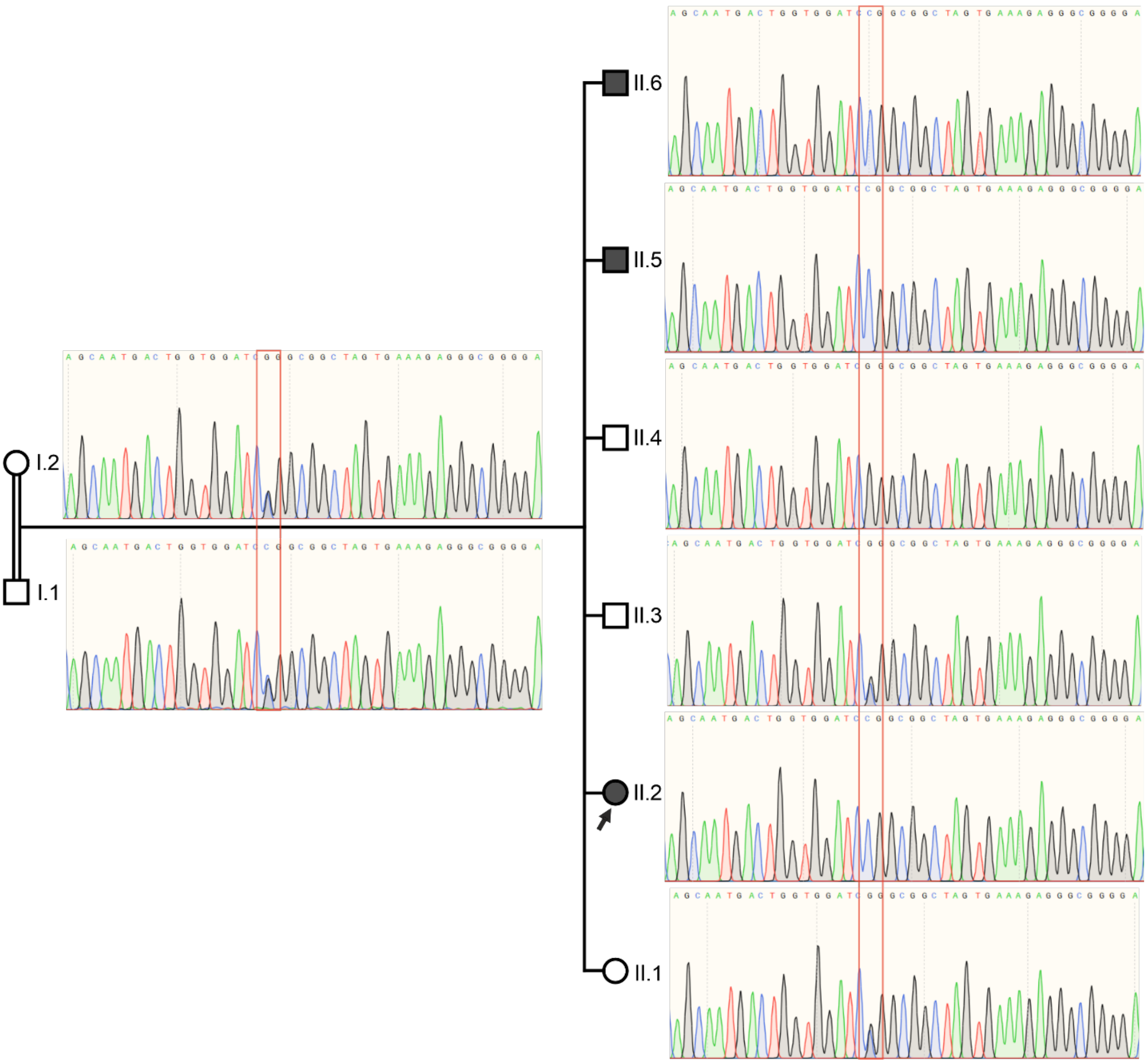

Supplementary Figure 2. Sequence analysis of all eight family members. Sanger sequencing confirmed the homozygous *CACNB3*:c.316G>C mutation in the index patient II.2 and her two affected brothers II.5 and II.6. Position 316 is highlighted by a red box. The pedigree of the family shows three affected members (II.2, II.5, II.6) suffering from IIN (filled symbols). The index patient (II.2) is marked by an arrow. Circles represent females, squares represent males.

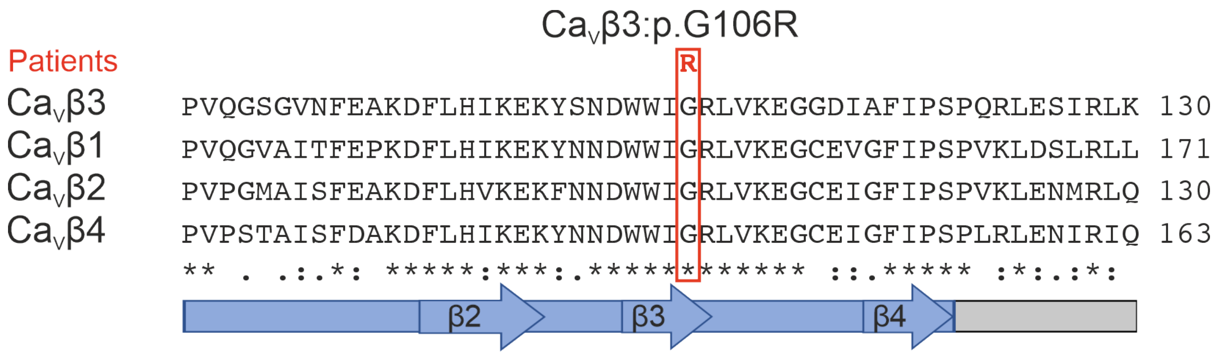

Supplementary Figure 3. Sequence alignment of all human Cavβ paralogs (Cavβ1-4). The mutation c.316G>C causes an exchange of a highly conserved glycine (G) to an arginine (R, red) in the third β-sheet (β3) of the SH3 domain. Secondary structure elements of Cavβ3 are drawn below the alignment. The SH3 domain is shown in blue with β-sheets depicted as arrows, and the HOOK region in grey. The position of the p.G106R missense mutation is highlighted by a red box. * (asterisk) – single, fully conserved residue; : (colon) – conservation between groups of strongly similar properties; . (period) – conservation between groups of weakly similar properties.

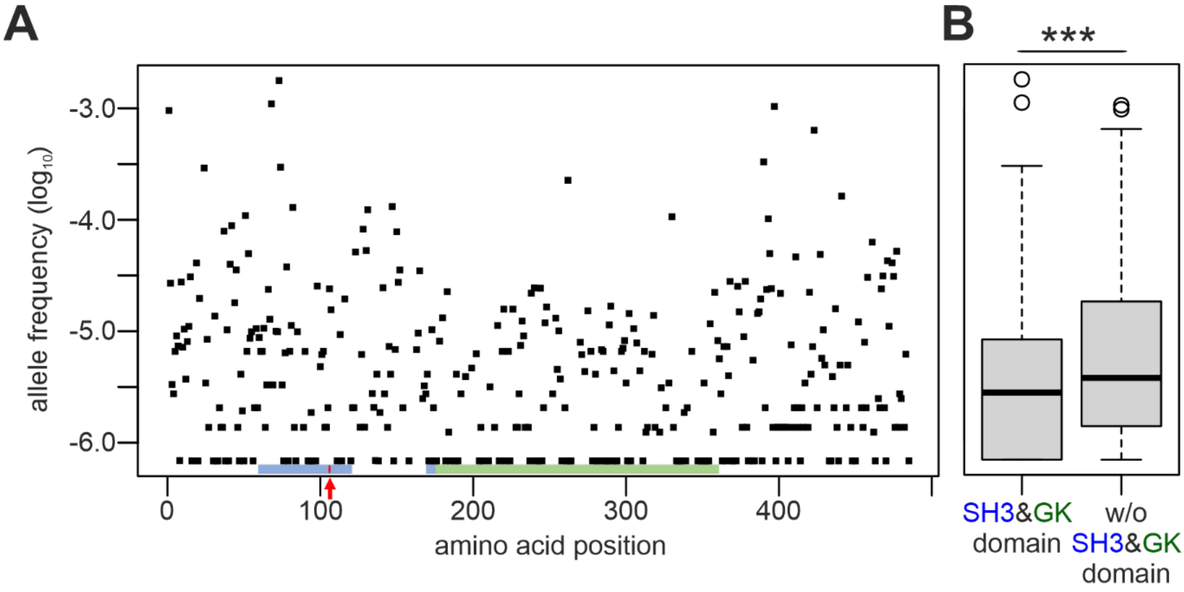

Supplementary Figure 4. Distribution of variant allele frequencies over the Cavβ3 protein sequence. (A) Allele frequencies of non-synonymous variants (y-axis) were summed up for each amino acid position (x-axis) and plotted as black squares. In total, non-synonymous variants were reported for 361 amino acid positions of the Cavβ3 sequence (from gnomAD). The positions of the SH3 and GK domain are indicated by blue and green, respectively. The position of the mutation is shown in red by an arrow. **(B)** Box plots of allele frequencies of variants at the SH3 or GK domain (N = 173) compared to the remaining part of the protein (without the SH3 and GK domain, N = 188). Allele frequencies at the SH3 and GK domain are significantly reduced (P = 0.0006745, W = 12894, Wilcoxon rank sum test with continuity correction). The black lines indicate the medians, the grey boxes show the interquartile ranges, the whiskers extend to maximally 1.5-fold interquartile range.

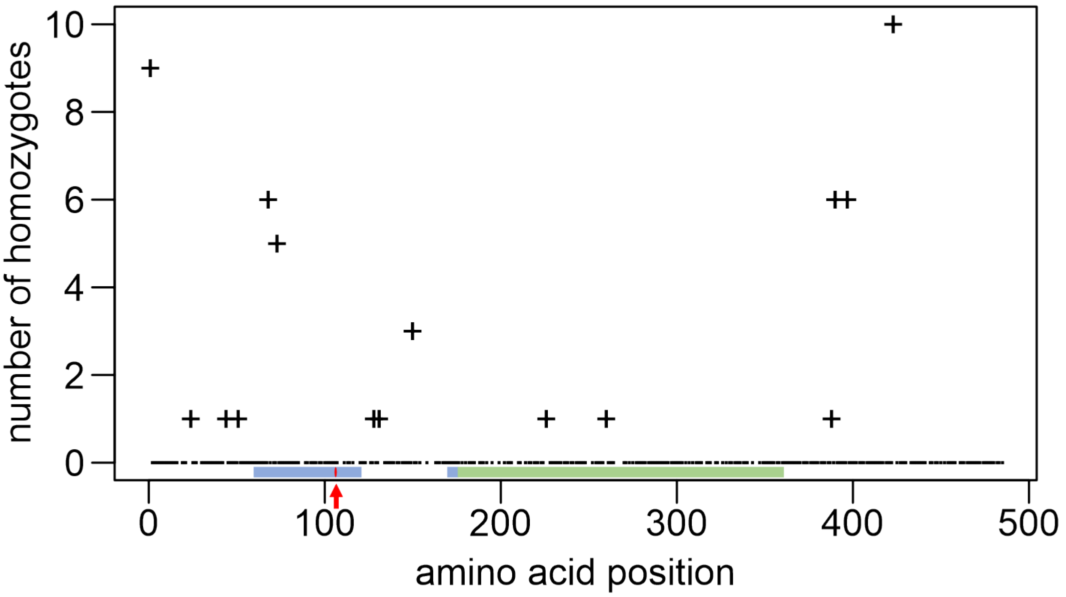

Supplementary Figure 5. Distribution of homozygous missense variants over the Cavβ3 protein sequence. The number of individuals carrying homozygous missense variants (y-axis) is shown for each amino acid position of the Cavβ3 sequence (x-axis) by a "+" symbol (in total 53 individuals with variants at 15 different loci, source: gnomAD). Homozygous nonsense/stop-gain variants were not present. The positions of the SH3 and GK domain are indicated by blue and green, respectively. The position of the mutation is shown in red by an arrow.

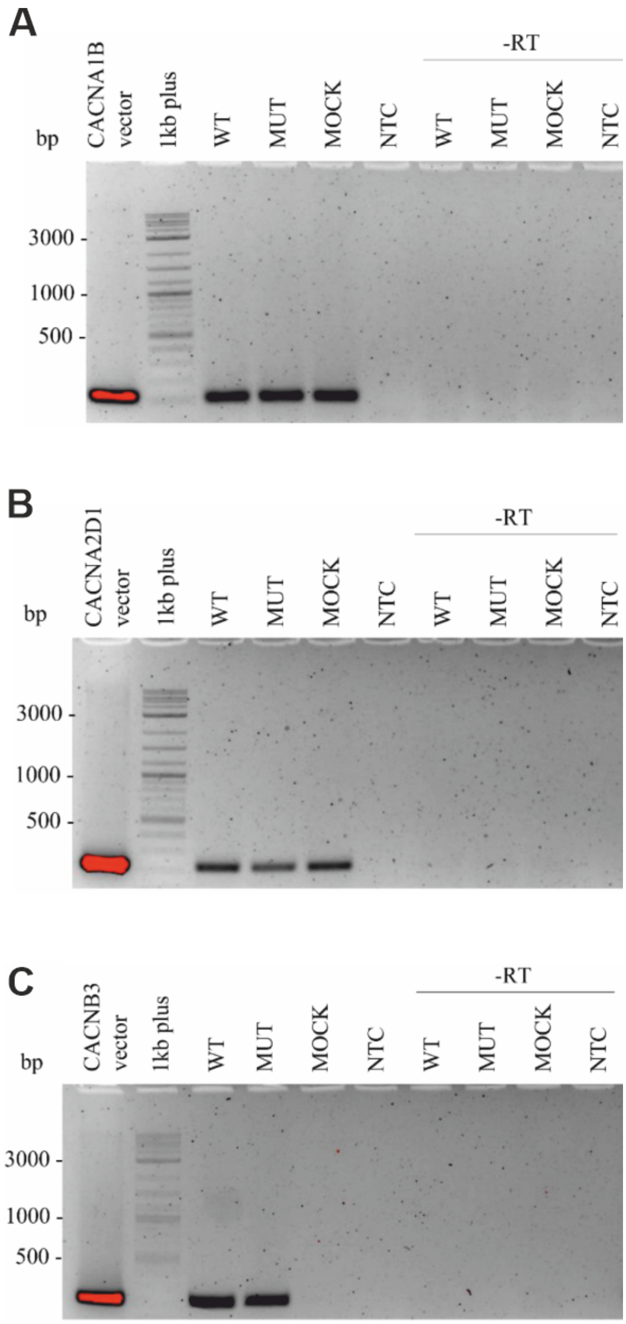

Supplementary Figure 6. Similar expression of VGCC subunits confirmed by RT-PCR. RT-PCR analysis of **(A)** *CACNA1B*, **(B)** *CACNA2D1*, and **(C)** *CACNB3* expression in transfected HEK293T cells used for Ca^{2+} live-cell imaging. Cells were co-expressing VGCC subunits with either *CACNB3* (WT) or *CACNB3* c.316G>C (MUT) or without *CACNB3* (MOCK). Negative control RT-PCR reactions lacking reverse transcriptase (-RT) or template (NTC) are shown on the right, positive control PCR is shown on the left. Red color indicates overexposure. NTC: non-template control; bp: base pairs.

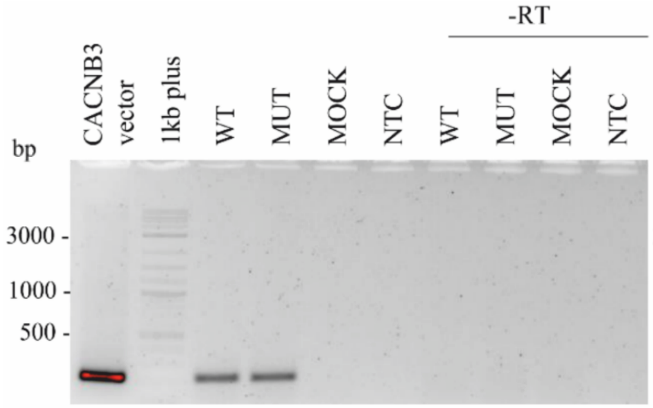

Supplementary Figure 7. RT-PCR confirms comparable wild-type and mutant Cavβ3 expression. RT-PCR analysis of *CACNB3* (WT), *CACNB3* c.316G>C (MUT), and mock (MOCK) transfected HEK293T cells used for Ca^{2+} live-cell imaging. Negative control RT-PCR reactions lacking reverse transcriptase (-RT) or template (NTC) are shown on the right, positive control PCR is shown on the left. Red color indicates overexposure. NTC: non-template control; bp: base pairs.

Supplementary Table 1. Variants in the coding sequences (±10 bases) of *CACNB3* (NM_000725.3) and *RAPGEF3* (NM_001098531.2).

**Gene**	**Exon**	**NT change**	**AA change**	**Zygocity**	**ExAC [%]**	**EVS [%]**	**dbSNP**
CACNB3	13	c.1267C>T	p.R423C	het	0.00826	N/A	rs111729272
CACNB3	13	c.1189C>T	p.R397W	het	0.1	0.13	rs140141253
RAPGEF3	13	c.1306G>A	p.A436T	het	0.1934	0.1462	rs141880710
RAPGEF3	17	c.1668T>C	p.V556V	het	0.3148	0.0077	rs145769431
RAPGEF3	19	c.1876C>T	p.L626F	het	N/A	N/A	N/A
RAPGEF3	21	c.2179G>A	p.V727M	het	0.0067	0.0154	rs371356808
RAPGEF3	26	c.2570G>A	p.S857N	het	0.01	0.0077	rs200517997
RAPGEF3	28	c.2655C>T	p.C885C	het	0.0607	0.03	rs146714167

ExAC: Exome Aggregation Consortium; EVS: Exome Variant Server; dbSNP: Single Nucleotide Polymorphism database

Supplementary Table 2. Plasmids used in this study.

**Plasmid**	**Gene**	**comment**
pcDNA3.1_CavBeta3_rat_wt	*CACNB3*, rat	Addgene #26574; kind gift by Diane Lipscombe
pcDNA3.1_CavBeta3_rat_mut	*CACNB3*:c.316G>C, rat	Generated using site directed mutagenesis
pcDNA3.1	none	mock
pcDNA6_Cav2.2_human	*CACNA1B*, human	Addgene #62574; kind gift by Diane Lipscombe
pcDNA3.1_Cavalpha2delta1_rat	*CACNA2D1*, rat	Addgene #26575; kind gift by Diane Lipscombe
pAAV_UbC-tdTOMATO	tdTOMATO	Addgene #62516; kind gift by Jae Lee and Pantelis Tsoulfas
mCherry-CD9-10	*mCherry-CD9-10* human	Addgene #55013; kind gift by Michael Davidson
GFP-CACNA1B	*GFP-CACNA1B* rabbit	Addgene #58737; kind gift by Annette Dolphin

Supplementary Table 3. Primer used in this study.

Name	Sequence
CACNB3_Rat_SDM_316_F	CAGCAATGACTGGTGGATCCGGAGGCTAGT GAAAGAAGG
CACNB3_Rat_SDM_316_R	CCTTCTTTCACTAGCCTCCGGATCCACCAGT CATTGCTG
CACNA1B_qPCR_Human_F	TCCACAAGGGCTCTTACCTG
CACNA1B_qPCR_Human_R	CCTCAGTGTTCGCAGGTC
CACNA2D1_Seq1_F	GCTGGCCTTGACTCTGACAC
CACNA2D1_qPCR_Rat_R	CAGTTGGCGTGCATTATTTG
CACNB3_Hu_Rat_Seq1_F	CCAAGCACAAACCTGTGGC
CACNB3_qPCR_H_Rat_R3	TGAGCCGGATGCTCTCCAG

Supplementary Table 4. LMM results for resting Ca^{2+} (related to Figure 4B).

condition	emmean	SE	df	lower.CL	upper.CL
mock	0.291	0.00336	10.09	0.284	0.299
wt	0.312	0.00362	13.49	0.305	0.320
mut	0.291	0.00326	8.93	0.284	0.299

emmean: estimated marginal mean; df: degrees of freedom; CL: 95% confidence level

Supplementary Table 5. *Post-hoc* results for resting Ca^{2+} (related to Figure 4B).

Contrast	estimate	SE	df	t.ratio	p.value
mock – wt	-0.021188	0.00315	1551	-6.725	<.0001
mock – mut	-0.000253	0.00269	1562	-0.094	0.9951
wt – mut	0.020935	0.00306	1535	6.844	<.0001

df: degrees of freedom

Supplementary Table 6. LMM results for Ca^{2+} peak amplitude (related to Figure 4C).

condition	emmean	SE	df	lower.CL	upper.CL
mock	0.0626	0.0149	6.85	0.0273	0.0979
wt	0.1511	0.0152	7.47	0.1157	0.1866
mut	0.0937	0.0147	6.62	0.0584	0.1290

emmean: estimated marginal mean; df: degrees of freedom; CL: 95% confidence level

Supplementary Table 7. *Post-hoc* results for Ca^{2+} peak amplitude (related to Figure 4C).

contrast	estimate	SE	df	t.ratio	p.value
mock – wt	-0.0885	0.00733	1552	-12.068	<.0001
mock – mut	-0.0311	0.00625	1548	-4.969	<.0001
wt – mut	0.0574	0.00711	1553	8.076	<.0001

df: degrees of freedom

Supplementary Table 8. LMM results for Ca^{2+} area under the curve (related to Figure 4D).

condition	emmean	SE	df	lower.CL	upper.CL
mock	12.8	3.05	7.87	5.8	19.9
wt	29.8	3.12	8.61	22.7	36.9
mut	18.0	3.00	7.33	11.0	25.0

emmean: estimated marginal mean; df: degrees of freedom; CL: 95% confidence level

Supplementary Table 9. *Post-hoc* results for Ca^{2+} area under the curve (related to Figure 4D).

contrast	estimate	SE	df	t.ratio	p.value
mock – wt	-16.94	2.00	1167	-8.462	<.0001
mock – mut	-5.14	1.79	1165	-2.880	0.0113
wt – mut	11.80	1.96	1162	6.035	<.0001

df: degrees of freedom

Supplementary Table 10. GLMM results for co-localisation (related to Figure 5B).

condition	emmean	SE	df	asymp.LCL	asymp.UCL
mut	-3.88	0.386	Inf	-4.64	-3.13
wt	-3.10	0.347	Inf	-3.78	-2.42

Results are given on the log odds ratio (not the response) scale. emmean: estimated marginal mean; df: degrees of freedom; LCL: lower 95% confidence level; UCL: upper 95% confidence level

Supplementary Table 11. *Post-hoc* results for co-localisation (related to Figure 5B).

contrast	estimate	SE	df	z.ratio	p.value
mut – wt	-0.787	0.271	Inf	-2.901	0.0037

Results are given on the log odds ratio (not the response) scale. df: degrees of freedom

Supplementary Table 12. LMM results for resting Ca^{2+} (related to Figure 6B).

condition	emmean	SE	df	lower.CL	upper.CL
mock	0.252	0.00312	11.14	0.245	0.259
wt	0.244	0.00298	9.36	0.237	0.250
mut	0.244	0.00289	8.26	0.238	0.251

emmean: estimated marginal mean; df: degrees of freedom; CL: 95% confidence level

Supplementary Table 13. *Post-hoc* results for resting Ca^{2+} (related to Figure 6B).

contrast	estimate	SE	df	t.ratio	p.value
mock – wt	0.008293	0.00206	1585	4.023	0.0002
mock – mut	0.007844	0.00197	1584	3.973	0.0002
wt – mut	-0.000449	0.00177	1585	-0.254	0.9651

df: degrees of freedom

Supplementary Table 14. LMM results for Ca^{2+} peak amplitude (related to Figure 6C).

condition	emmean	SE	df	lower.CL	upper.CL
mock	0.375	0.0224	9.00	0.324	0.425
wt	0.358	0.0218	8.16	0.308	0.408
mut	0.407	0.0215	7.62	0.357	0.457

emmean: estimated marginal mean; df: degrees of freedom; CL: 95% confidence level

Supplementary Table 15. *Post-hoc* results for Ca^{2+} peak amplitude (related to Figure 6C).

contrast	estimate	SE	df	t.ratio	p.value
mock – wt	0.0166	0.01110	1583	1.494	0.2939
mock – mut	-0.0320	0.01060	1584	-3.006	0.0076
wt – mut	-0.0486	0.00954	1584	-5.093	<.0001

df: degrees of freedom

Supplementary Table 16. LMM results for Ca^{2+} area under the curve (related to Figure 6D).

condition	emmean	SE	df	lower.CL	upper.CL
mock	8.92	0.707	12.14	7.38	10.5
wt	9.46	0.670	9.87	7.96	11.0
mut	10.27	0.645	8.51	8.80	11.7

emmean: estimated marginal mean; df: degrees of freedom; CL: 95% confidence level

Supplementary Table 17. ***Post-hoc*** **results for Ca^{2+} area under the curve (related to Figure 6D).**

contrast	estimate	SE	df	t.ratio	p.value
mock – wt	-0.534	0.503	1519	-1.061	0.5384
mock – mut	-1.344	0.481	1518	-2.797	0.0145
wt – mut	-0.810	0.430	1519	-1.884	0.1437

df: degrees of freedom
